# A Randomized Watermarking Technique for Detecting Malicious Data Injection Attacks in Heterogeneous Wireless Sensor Networks for Internet of Things Applications

**DOI:** 10.3390/s18124346

**Published:** 2018-12-09

**Authors:** Arwa Alromih, Mznah Al-Rodhaan, Yuan Tian

**Affiliations:** 1 Information Systems Department, King Saud University, Riyadh 12371, Saudi Arabia; 2 Computer Science Department, King Saud University, Riyadh 12371, Saudi Arabia; rodhaan@ksu.edu.sa (M.A.-R.); ytian@ksu.edu.sa (Y.T.)

**Keywords:** Internet of Things (IoT), wireless sensor network (WSN), data integrity, watermark, data injection attack

## Abstract

Using Internet of Things (IoT) applications has been a growing trend in the last few years. They have been deployed in several areas of life, including secure and sensitive sectors, such as the military and health. In these sectors, sensory data is the main factor in any decision-making process. This introduces the need to ensure the integrity of data. Secure techniques are needed to detect any data injection attempt before catastrophic effects happen. Sensors have limited computational and power resources. This limitation creates a challenge to design a security mechanism that is both secure and energy-efficient. This work presents a Randomized Watermarking Filtering Scheme (RWFS) for IoT applications that provides en-route filtering to remove any injected data at an early stage of the communication. Filtering injected data is based on a watermark that is generated from the original data and embedded directly in random places throughout the packet’s payload. The scheme uses homomorphic encryption techniques to conceal the report’s measurement from any adversary. The advantage of homomorphic encryption is that it allows the data to be aggregated and, thus, decreases the packet’s size. The results of our proposed scheme prove that it improves the security and energy consumption of the system as it mitigates some of the limitations in the existing works.

## 1. Introduction

The Internet of Things (IoT) is an ecosystem that interconnects “smart” objects that are connected to the internet [1]. These objects sense data from the environment and route it to the internet to be collected, processed, and analyzed using technologies [2]. The IoT architecture is generally composed of three layers: the application layer, the network layer, and the perception layer. The perception layer has all of the physical objects that interconnect together and form a network [3]. Wireless Sensor Networks (WSN) are considered one of the main enabling technologies for this layer [3,4]. They are composed of low-power sensor nodes that have limited computational and storing capabilities and a powerful base station (BS) [5]. Wireless sensor networks are used in a wide range of applications, such as environmental monitoring and control, health care systems, office automation, and home control [3,6]. In these applications, sensors share their data through the shared wireless medium [3]. Due to this reason and the limitation detailed earlier, they can be vulnerable to multiple numbers of security attacks that can affect the integrity and confidentiality of the data, such as a packet drop, data modification, a packet replay, and false data injections [7,8]. By injecting false data into the network, the attacker can make fake reports that will lead to serious damage or dangerous effects. Therefore, it is very crucial to have a countermeasure to filter false data as early and accurately as possible.

Some mechanisms have been developed to secure the IoT from such attacks. These solutions vary regarding techniques [1]. Some of these solutions use software attestation, anomaly detection techniques, trust-based techniques, signature-based techniques, or watermarking techniques. However, most of these mechanisms have their limitations and cannot be used effectively to provide both confidentiality and integrity. Software attestation techniques require special hardware. The anomaly detection and trust-based techniques can suffer from a high rate of false positives [9]. They also involve extra steps of building a distribution of the normal behavior first. Signature-based techniques can be considered computationally complex [10]. Watermarking techniques are used in many applications. They were originally used to protect multimedia content and relational databases [11]. Many researchers have used them to secure sensory data. In the watermarking process, each datum is embedded with a unique watermark by the sensor node before sending it. The access point can then verify its integrity. The above weaknesses of these techniques make them complex [12], computationally expensive, and some are application- or context-dependent [9]. Therefore, research effort should be made to design a new low-complexity scheme, yet secure data integrity system that can detect and filter out false data injection attacks.

The main contribution of this paper is the offer of a Randomized Watermarking Filtering Scheme (RWFS) for WSN, which can filter false injected data effectively and as early as possible without relying on any static node location or secure routing protocols. The proposed system aims to protect the integrity, authenticity, and confidentiality of sensory data using a lightweight watermarking technique. It will provide en-route rather than end-to-end filtering to minimize communication overhead by reducing the number of false data reports. Integrity and authenticity will be provided by embedding a watermark at random locations within the data. Embedding a watermark within multiple random locations will make it more difficult for an adversary to inject a false report. The system will also use homomorphic symmetric encryption to provide confidentiality.

The main purpose of this paper is to address the following issues:

(1) Develop a novel energy-efficient scheme that aims to minimize both the packet size and number of communications between nodes.
(2) Design a new and random way of embedding the watermark that will be based on pseudorandom number generator (PRNG) algorithm.
(3) Investigate and evaluate the security and performance of the proposed watermark technique in filtering malicious data injection attacks.

The rest of this paper is organized as follows. Section 2 reviews related work by other scholars using different approaches. We address the different system models and assumptions in Section 3, and we present the proposed scheme in detail in Section 4. Section 5 presents a security analysis of the proposed scheme. Section 6 describes the performance evaluation methodology and results from the scheme simulation. A summary of the contributions of this work and a discussion of possible future work are presented in Section 7.

## 2. Literature Review

Several researchers have conducted surveys on the security-related issues in the IoT [1,13,14]. These surveys provide a comprehensive understanding of the existing security vulnerabilities and challenges that different applications face. Many recent types of research have provided countermeasures against data injection attacks in the IoT. These countermeasures can be divided into five main categories: software attestation, anomaly detection, trust management, signature-based techniques, and digital watermarking techniques. These scholars have employed solutions to counter injection attacks on the sensory data of IoT applications.

Numerous papers have been proposed using a software attestation technique that looks for evidence of node modification. The main idea of software attestation techniques is to challenge nodes with a challenge request where they need to respond with the correct solution in order to check the node software’s integrity. Asokan et al. have proposed SEDA, a collective software-based attestation solution that is based on a challenge–response mechanism [15]. SEDA forms the network as a spanning tree where each child node (attester) is challenged by its parent (verifier) to respond with the correct report that is calculated based on a nonce and a shared secret. SEDA only considers software-based attacks; therefore, it cannot secure networks in the presence of a physical attacker [16].

Sun et al. [17] have proposed an anomaly-detection-based mechanism that uses an Extended Kalman Filter (EKF) with the Generalized Likelihood Ratio (GLR) to check whether or not the new reading differs from the normal model’s state. The system starts by estimating the normal model of the network, and then a similarity check based on EKF and GLR will be performed on new measures that are received from neighbor nodes. Using anomaly detection techniques can be easily deployed on top of any application required. The only drawback of such techniques is that they need to accurately define the boundary for expected behavior [9].

Location-aware End-to-end Data Security (LEDS) [7] proposed a location-based trust management technique that relies on the location of the nodes. LEDS ensures data integrity and authenticity using a location-aware key management framework where three different types of keys are generated based on the location of the sensors. The weakness of this work is that it relies on a static, configured location parameter, which limits its usage to a specific number of applications [18].

Signature-based detection techniques have been the basis of much more research. In the Dynamic En-route Filtering scheme (DEF) scheme [19], a message authentication code (MAC) is used as a signature to verify the integrity of a report. Each sensing node will generate multiple authentication keys using one-way hash chains. These keys will be disseminated by the cluster head (CH) to the forwarding nodes using preloaded secret keys. DEF uses many keys, since it involves using both authentication keys and secret keys. It also involves a vast number of communication messages between nodes to complete a single round.

Di Pietro et al. [20] and Kumar et al. [21] have used a keyed-hash message authentication code (HMAC) to provide integrity. Cui, et al. [22] proposed a new scheme to provide both integrity and confidentiality. To ensure the end-to-end integrity of aggregated data, they have used a homomorphic MAC (H-MAC) algorithm. The homomorphic MAC can be added, i.e., H−MAC(a+b) = H−MAC(a) + H−MAC(b). Moreover, a homomorphic encryption algorithm is used in this scheme to provide confidentiality. These schemes provide integrity at an additional cost of data processing and communication.

PCREF, which was proposed in [18], uses a message authentication polynomial (MAP) to provide integrity. Every node is assigned a polynomial primitive that is used for authentication and checking. After a node has sensed some data, it will generate a message authentication polynomial (MAP) and append it to the measurement to be forwarded to the base station. The scheme only provides end-to-end integrity as it evaluates the integrity at the BS.

IA-CTR [23] is an integrity-aware conventional counter mode that is a variant of the conventional counter mode. This mode can provide message integrity and privacy through using a shared counter during block encryption algorithm. This technique does not actually require any additional computational overhead, but using counter mode encryption is not recommended as it is malleable, i.e., the attacker can manipulate the ciphertext so that it decrypts to any message of his choice [24].

Another type of integrity mechanism is using watermarking techniques. There are three types of digital watermarks: robust, fragile, and semi-fragile watermarks [25]. The first two types have been the subject of research in wireless sensor networks. Semi-fragile watermarking is not typically used in WSNs. Work on using watermarking techniques for integrity and authentication in WSNs began in 2003. Feng et al. [26] proposed the first watermarking system that embeds cryptographically encoded signatures into data.

Fragile watermarking is used to provide data integrity authentication through embedding a watermark into the original data. Tiwari et al. [27] have developed a hop-by-hop fragile watermarking technique. They have assumed the network to be homogeneous and formed as a tree with each cluster head to have at most three children. The technique uses data aggregation to reduce communication overhead. The watermark size is only one bit, and the generation process is very simple by using the exclusive or (XOR) operator. The embedding is done at the least significant bit of the data. The technique is simple but not secure. A cross-layer watermarking-based data aggregation (CLWDA) [28] is another fragile watermarking mechanism that adopts two types of watermarks. The first is called a fragile watermark (FW), which is used to validate data sent from the first level of the network. The higher level of the network (aggregator nodes) uses a reinforced fragile watermark (RW), which is more secure as the watermark is encrypted using the asymmetric key. The mechanism adopts a different embedding approach than most of the proposed schemes. The embedding position of the watermark will be dynamic, and it will be calculated based on the wakeup interval used by each node. Although this makes it somewhat random, it can be easily detected as it will form a pattern. The CLWDA mechanism also requires synchronization between nodes to determine wake-up times.

A watermarking algorithm based on a one-way hash function was proposed by Sun et al. [29]. The watermark is computed for each byte of data, and then grouped using the XOR function. The final watermark is then divided between the bytes and embedded in predefined redundant spaces. The proposed algorithm is computationally simple. However, it only provides end-to-end integrity.

Hameed et al. [30] had proposed a zero-watermarking technique (ZWT) that generates a watermark from the properties of the, sensed data. After encrypting the watermark using a shared secret key, the sensor embeds the encrypted watermark at the end of the sensed data before it is sent to the base station. ZWT was tested against other watermarking techniques, and the results showed that it does not introduce any computation overhead as the generation process is simple. ZWT is vulnerable to many security breaches in the case of exposing the shared secret key as it uses only one key for the whole network.

Watermarking-LEACH [31] is an integrity-preserving approach that adds a watermark on top of the LEACH routing protocol. It modifies the original approach of LEACH by adding watermarking embedding at the cluster heads and watermark extraction at the BS. This approach involves minimal modification to a very well-known routing protocol. However, it only provides integrity at one part of the communication, which is between cluster heads and BS.

A semi-blind watermarking technique that uses linear interpolation to embed the watermark into the data was proposed by Lalem et al. [25]. The main advantage of the technique is that it does not introduce any extra bits for the watermark. However, it uses a fixed watermark parameter for all nodes in the system that can be easily cracked.

Robust watermarking, which is used to provide copyright protection, was combined with the fragile watermark by Ren et al. [32]. The authors proposed a digital watermarking scheme based on multiple functions to protect images transmitted in a WSN. The scheme embeds two types of watermarks (robust and fragile watermarks) to provide copyright protection and integrity, respectively. The robust watermark is generated from the stable features of the original picture. Image pixels are used to generate the fragile watermark. The scheme is only used in wireless multimedia sensor networks that deal with visual sensors.

Guan et al. [33] proposed another robust watermarking scheme for node authentication. The proposed scheme computes the watermark based on a randomly chosen data sampling time interval. The scheme is not robust in detecting false data insertion attacks, as it does not consider all of the data in generating the watermark.

Reversible watermarking techniques were proposed in [34,35,36]. Shi et al. [34] proposed a reversible watermarking algorithm based on prediction-error expansion. The algorithm groups every two adjacent data items together. The first one is used to generate the watermark, while the other is used as a carrier for it. The drawback of this method is that verification is end-to-end, since verification needs extra storage as it uses buffering.

Ding et al. [35] proposed a similar end-to-end algorithm (called RDE), in which the watermark is generated based on the difference expansion. The algorithm groups n data items and uses them all to calculate the watermark. The embedding process calculates the weighted difference between each data item with the first data item. The embedding process is straightforward, as every bit of the watermark is embedded in the least significant bit of each data item. This algorithm can ensure the complete recovery of data at the base station. However, it cannot detect integrity along the route of the communication.

Li et al. [36] have proposed an improved version of the difference expansion method used by Ding et al. [35]. The whole network was assumed to be viewed as an image with each sensor node as a pixel. The technique generates a single watermark for the all of the system’s data and splits it into several segments. Each segment is assigned to random sensors for embedding. The extracting operation will use a location map to know which sensor nodes were chosen to embed the watermark. The weakness of this technique is that responsibility for the watermarking process in each round is given to a single node.

## 3. System Models and Assumptions

### 3.1. Network Model

**Figure 1:**
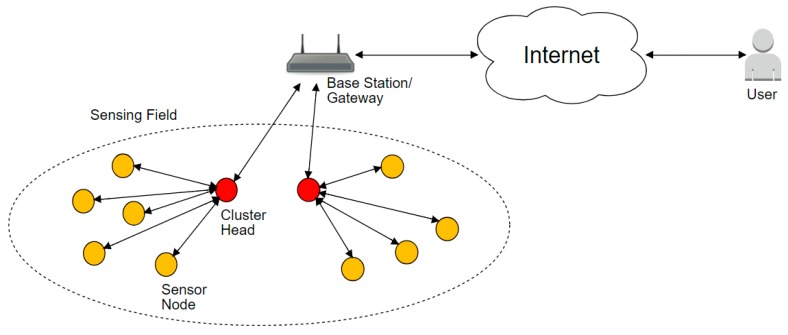
The network model.

The network will be assumed to consist of N nodes that are uniformly randomly distributed within an M×M square area. Nodes are heterogeneous in terms of energy, i.e., there are two types of nodes: normal nodes and advanced nodes. Normal nodes have energy $E_{0}$, while advanced nodes have a times more energy than normal nodes $E_{0}\left(1+a\right)$. This will mean that some nodes are equipped with more energy than other nodes. Sensor nodes are formed into multiple clusters. Clusters will each have n sensing nodes and a cluster head CH. These two types of nodes are shown in Figure 1. Nodes that belong to the same cluster will know the IDs of each other and can communicate with each other. Routing the data to the base station is done through the *CH* nodes.

### 3.2. Clustering Model

Clustering is used as a routing protocol in the network. Cluster-based routing is an efficient approach to maintain a system’s scalability, lifetime, and energy efficiency within a cluster. It enables clusters to perform data aggregation in order to decrease the number of transmitted messages to the access point (AP). In our schemed, clusters are formed using the Enhanced Distributed Energy Efficient Clustering (E-DEEC) [37] protocol, where cluster head selection is made based on nodes’ residual energy. E-DEEC uses a three-level heterogeneous network concept, where the system has three types of nodes: normal nodes, advanced nodes, and super nodes. Each type of node has its own initial energy. Normal nodes have $E_{0}$ energy. Advanced nodes have a times more energy than normal nodes, i.e., $E_{0}\left(1+a\right)$. Super nodes have b times more energy than the normal ones, i.e., $E_{0}\left(1+b\right)$.

In E-DEEC, any node can be chosen to be a cluster head. A node can be selected as a *CH* if it has more residual energy than the total average energy of the network. This way, we will ensure that super nodes and advanced nodes are not exhausted, and, hence, it prolongs the lifetime of the network. Cluster heads are chosen based on a probability value $p_{i}$ that is computed differently based on the node’s type. $p_{i}$ can be computed as follows:

(1) $$p_{i}=\{\begin{array}{cc} \frac{p_{opt}E_{i}\left(r\right)}{\left(1+m\left(a+m_{0}b\right)\right)\bar{E}\left(r\right)} & if\;s_{i}\;is\;a\;normal\;node\\ \frac{p_{opt}\left(1+a\right)E_{i}\left(r\right)}{\left(1+m\left(a+m_{0}b\right)\right)\bar{E}\left(r\right)} & \;if\;s_{i}\;is\;an\;advanced\;node\\ \frac{p_{opt}\left(1+b\right)E_{i}\left(r\right)}{\left(1+m\left(a+m_{0}b\right)\right)\bar{E}\left(r\right)} & if\;s_{i}\;is\;a\;super\;node \end{array}$$

where $p_{opt}$ is the probability of the optimum number of cluster heads set by the network administrator, $E_{i}\left(r\right)$ is the remaining energy of node *i* of the *r*th round, *m* and $m_{0}$ are the fractions of the number of advanced nodes and super nodes, respectively, *a* and *b* are the energy enhancement levels of advanced nodes and super nodes, respectively, and $\bar{E}\left(r\right)$ is the average energy of the network at the *r*th round.

### 3.3. Radio Energy Dissipation Model

**Figure 2:**
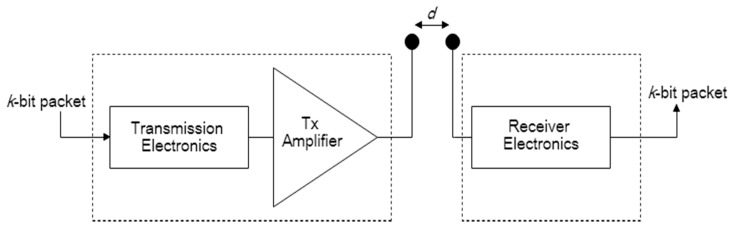
The radio energy model.

To analyze the performance of our proposed protocol, we need to know the amount of energy that is consumed by a sensor node. Therefore, we use a similar Radio Energy Model to the one proposed in [38]. According to [38], the radio energy dissipation model is illustrated as in Figure 2.

In this model, both the free space ($d^{2}$ power loss) and the multipath fading ($d^{4}$ power loss) channel models are used. If the distance is less than a threshold $\;d_{0}$, the free space model is used; otherwise, the multipath model is used. Thus, to transmit a k bit packet over a distance d, the consumption energy is computed as [38]:

(2) $$E_{Tx}\left(k,d\right)=\;\{\begin{array}{c} k\times E_{elec}+k\times E_{fs}\times d^{2}\;\;\;\;\;\;if\;d<\;d_{0}\\ k\times E_{elec}+k\times E_{mp}\times d^{4}\;\;\;\;\;\;\;if\;d\;>\;d_{0} \end{array}$$

and to receive a k bit packet, the consumption energy is [38]:

(3) $$E_{Rx}\left(k\right)=k\times E_{elec}$$

where $E_{elec}$ denotes the electronics energy, which depends on factors such as the digital coding, modulation, filtering, and spreading of the signal. $E_{fs}$ is the free space power loss and $E_{mp}$ is the multipath fading loss. The value of threshold distance $d_{0}$ is given by $d_{0}=\sqrt{\frac{E_{fs}}{E_{mp}}}$.

From Equations (2) and (3), it is clearly shown that the energy consumed for data transmission increases as the packet size increases and the square or biquadratic of distance, while the energy consumed for reception is only proportional to the packet size [39].

### 3.4. Adversary Model

In this paper, we consider an adversary model that is similar to the one used in [22,40]. The model has three types of adversaries:

(1) **A passive adversary** that can observe and obtain packets by eavesdropping on a network transmission.
(2) **An external adversary** who can originate and inject false data from the outside. He can also replay old packets or modify the transmitted data.
(3) **An internal adversary** that can physically compromise sensor nodes or cluster heads.

Each one of the three types of adversaries can launch a different number of attacks based on his abilities.

An adversary of **Type 1** can obtain secret information by eavesdropping.

(a) **Eavesdropping Attack:** this attack concerns the passive adversary who aims to obtain information by listening to the message transmission in the broadcasting wireless medium [41].

The adversary of **Type 2** can send false information by modifying the content of packets, injecting false data, or replaying old messages. Therefore, he can launch three kinds of attacks:

(a) **Injection Attack:** In this attack, an adversary injects false data into the network and compromises the trust in the communicated information.
(b) **Replay Attack:** the attacker captures some packets and resends them at a future time.
(c) **Modification Attack:** the adversary modifies the data without knowing the content.

Lastly, the adversary of **Type 3** can sense and know all secret parameters. In this situation, he can launch a compromise attack.

(a) **Node Compromise Attack:** the attacker physically comprises a node or CH to obtain secret information and generate the secret parameters of the network.

## 4. Proposed Scheme

**Figure 3:**
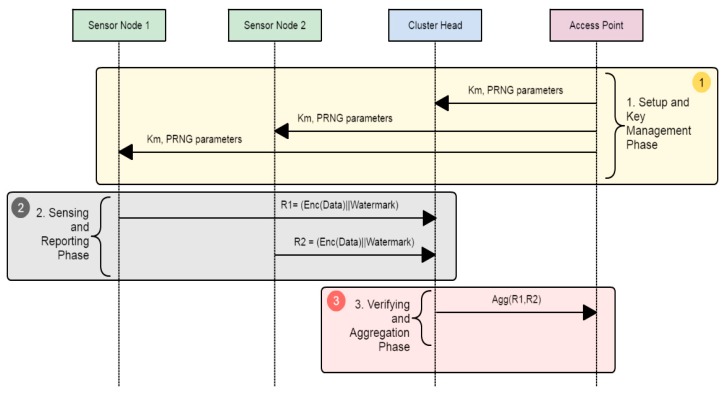
The sequence diagram of the proposed scheme.

The proposed system provides data integrity, authenticity, and confidentiality by embedding a watermark at random locations within the data and using symmetric encryption. The proposed system consists of three phases as shown in Figure 3: (1) *Setup and Key Management*; (2) *Sensing and Reporting*; and (3) *Verifying and Aggregation*.

In the S*etup and Key Management* phase, the clusters are formed, and all authentication information is assigned to the sensors. After that, in the case of an event or based on a periodic interval, sensor nodes will start to sense information, encrypt it, embed the watermark at a random place, and then send the final report *R_i_* to the cluster head to start the verification step. In the *Verifying and Aggregation* phase, the cluster head checks whether the received report is valid or not to filter bogus packets. The verification is done by regenerating the watermark from the extracted data. For each valid *R_i_* of the received reports, *CH* aggregates the sensed data, embeds a new watermark in the aggregated data, and forwards it to the access point. The AP individually decrypts and verifies the reports of each cluster, as it is assumed that the AP has all of the necessary keys and parameters.

Following this, the phases of RWFS are described in detail. All of the notation that is used later is listed in Table 1.

### 4.1. Phase 1: Set up and Key Management

In the set up step, the network uses the E-DEEC clustering algorithm to cluster the network into *j* clusters. In each cluster *j*, n sensing nodes and a CH will be assigned an ID. These nodes can communicate with the CH directly. After that, the AP initializes each node with the necessary set up parameter by sending the following $\left\{m_{j},\;c_{j},\;a_{j},\;H\left(,\right),\;k_{m}\right\},$ where $K_{m}$ is the master key of the system that is used by each sensing node in the following function to produce the cluster key $K_{c}$

(4) $$K_{cj}=k_{m}⨁CH_{j}ID\;|\;H\;\left(K_{m}\right)$$

where $m_{j},\;c_{j},\;\mathrm{and}\;a_{j}$ are the parameters that are used for the pseudo-random number generator function. These parameters differ among clusters.

### 4.2. Phase 2: Sensing and Reporting

Each sensing node $S_{i}$ measures the data that it monitors from the environment as $D_{i}$ and then encrypts it as the following: $\;E_{i}=\;Enc\left(D_{i}K_{cj}\right)$. After that, the node generates the watermark of the encrypted measure as $W_{i}$. The embedding position of the watermark $W_{i}$ is randomly chosen and varies frequently. The position of each nibble (4 bits) of $W_{i}$ is generated using a PRNG. The PRNG is used to generate three random numbers for three different positions. Finally, after embedding the watermark, the report is sent as the following format to $CH_{j}$

(5) $$R_{i}=(E_{i}\;|\;W_{i}\;|\;Time)$$

The detailed procedure of Phase 2 is shown in Algorithm 1. 

**Algorithm 1** Sense and report algorithm (*m_j_*,*c_j_*,*a_j_*,*k_cj_*)**Input:** pseudo random number generator parameters *m_j_*,*c_j_* and *a_j_*. Cluster key *k_cj_* **Output:**
*R_i_* = *E_i_W_i_*||*Timestamp* **1** *D_i_* = sensed data **2** *E_i_* = Enc(*D_i_*, *k_cj_*) **3** *W_i_* = HMAC(*E_i_* ||*S_i_* ||*Timestamp*) || **4 for**
*count*=1 *to numberOfPositions*
**do**
**5**


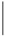

*X_n_*_+1_ = (*a_j_ X_n_* + *c_j_*) mod *m_j_*
**6**
*pos*[*count*] = *X_n_*_+1_**7 end** **8** *E_i_W_i_* = Embed(*W_i_*, *E_i_*, *pos*[])  ▷Embed watermark *W_i_* in *E_i_* at positions *pos*[] **9** *R_i_* = *E_i_W_i_* ||*Timestamp* **10** *Send*(*R_i_*, *CH_j_*)

#### 4.2.1. Encryption Algorithm

As mentioned in the introduction, the scheme uses a homomorphic encryption algorithm to encrypt data so that it can be aggregated. Moreover, since encryption algorithms are usually expensive and complex to compute, we have used a very lightweight encryption algorithm that was proposed by Castelluccia et al. [42]. The encryption function is as follows c = Enc(d, k, M) = d + k(modM), where *d* is the data to encrypt, *k* is the secret key for the node, and *M* is the modulus.

#### 4.2.2. Watermarking Algorithm

The primary goal of the proposed watermarking algorithm is to randomly embed the watermark within the data and still manage to extract it. Also, RWFS strives to resolve and effectively maintain the energy level of the nodes. The algorithm consists of two procedures: watermark generation and watermark embedding.

The watermark generation process takes sensory data after encryption $E_{i}\;$ as input from the sensor node $S_{i}\;$ and generates a watermark $W_{i}$. The time and sensor *ID* are also used as inputs to a keyed-hash message authentication code (HMAC) to produce the final watermark. The final watermark is calculated as:

(6) $$W_{i}\;=Hash\left(\left(E_{i}||\;Time\;||\;S_{i}ID\right),\;k_{cj}\right)$$

**Figure 4:**
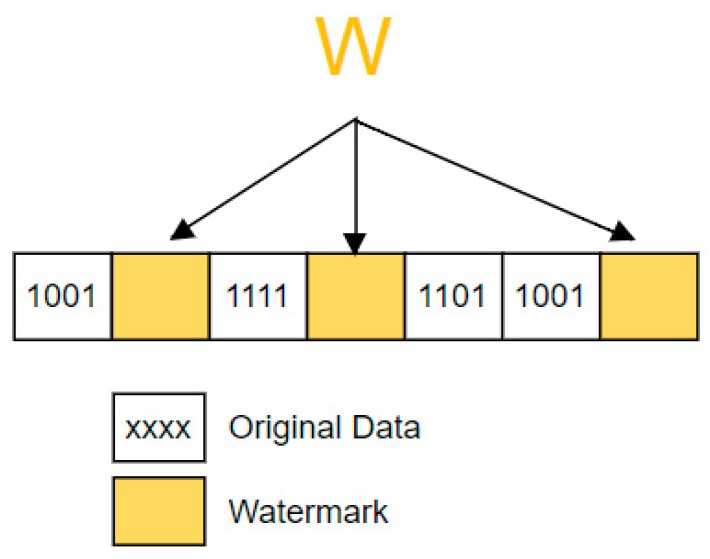
Watermark embedding.

After that, the watermark is embedded at random redundant spaces of the encrypted data $E_{i}$ as shown in Figure 4. These redundant spaces are determined by using a pseudo-random number generator.

There are many ways to build pseudo-random generators (PRNG) suitable for use with our system. For simplicity, we take the linear congruential generator (LCG) [43] in the following equation to compute each position of $W_{i}$

(7) $$X_{n+1}=\left(a_{j}X_{n}+c_{j}\right)mod\;m_{j}.$$

Here, $m_{j}>0$ is a modulus, $a_{j}\;(0<a_{j}<m_{j})$ is the multiplier, $c_{j}\;\left(0\leq c_{j}<m_{j}\right)$ is the increment, and $X_{n}\;\left(0\leq X_{n}<m_{j}\right)$ is the current seed. Each generated $X_{n+1}$ is used as a pseudo-random number and becomes the new seed. Each node computes three values of $X_{n+1}$ to use as the positions of $W_{i}$.

To avoid every cluster having the same number generated for it, each cluster *j* has different parameters for its pseudo-random number generator.

**Figure 5:**
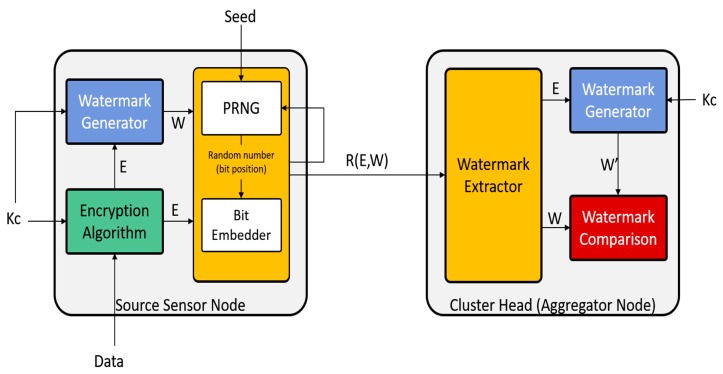
A block diagram of the Randomized Watermarking Filtering Scheme (RWFS) showing the modules of the sensor node and cluster head. It also shows the flow of data between the modules.

The sensor’s modules responsible for the two sub procedures of data encryption and watermarking generation are shown in Figure 5.

### 4.3. Phase 3: Verifying and Aggregation

After receiving reports from the sensing nodes in cluster *j*, *CH_j_* starts checking and verifying the integrity of each received report *R_i_*. It needs to check the embedded watermark and tries to regenerate it by using the shared secret key $K_{cj}$. Therefore, the verification phase relies on three conditions:

(1) The freshness of the timestamps Time attached to each report.
(2) Verifying the watermark embedded within the measurement.
(3) Having T or more reports where *T* is the number of sensor nodes belonging to cluster *j*.

Cluster heads can know whether or not the report is the newest measurement based on the field Time. This helps in detecting replayed false reports. For condition 2 and 3, the cluster head first extracts the watermark by generating the same number of random numbers to know exactly where the watermark was embedded. Next, it extracts $W_{i}$ from the received $R_{i}$ to obtain *E_i_*. A new watermark $W^{\prime }$ is generated from the value of *E_i_* received. If $W_{i}$ and W' are the same, the report is accepted. After that, $CH_{j}$ will have to wait for T reports. After receiving T reports, $CH_{j}$ aggregates the measurements received to obtain *agg_j_*, generates $\;W_{j}$, embeds it, and then sends it to the AP as $R_{j}\;=\;(agg_{j}\;|\;W_{j}\;|\;Time)$. Algorithm 2 details the steps of Phase 3.

**Algorithm 2** Verify and aggregate algorithm (R_i_)**Input:** reports R_i_ from sensor nodes in the cluster **Output:** R_j_ = agg_j_W_j_||Timestamp **1 *if***
*Timestamp is not fresh **then** Reject packet*; **2 *for***
*count=1 to numberOfPositions **do***
**3**


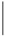


*X_n+1_ = (a_j_ X_n_ + c_j_) mod m_j_*

**4**

*pos[count] = X_n+1_*
**5** ***end*** **6** *E_i,_ W_i_ = Extract(E_i_W_i_, pos[])*  ▷*Extract watermark W_i_ and E_i_ from E_i_W_i_ at positions pos[]* **7** *W’ = HMAC(E_i_ ||S_i_ ||Timestamp)* **8 *if***
*W’ == Wi **then***
**9**


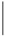


*Accept packet*

**10**

*agg_j_ = aggregate(E_i_)*
**11  *else*** **12** 
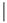
*Reject packet* **13 *end*** **14** *W_j_ = HMAC(agg_j_ || CH_j_ID ||Timestamp)* **15** *agg_j_W_j_ = Embed (W_j_, agg_j_, pos[])* **16** *R_j_ = agg_j_W_j_ ||Timestamp* **17** *Send(R_j_, AP)*

## 5. Security Analysis

In this section, we analyze the confidentiality and integrity properties of our proposed scheme for the adversary model presented in Section 3.4.

**Theorem** **1.** *Our scheme is robust against an adversary of **Type 1** and his attack as it provides end-to-end confidentiality of data*.

**Proof** **of** **Theorem** **1.** In the proposed scheme, all data transmitted from sensor nodes to the access point are encrypted with a homographic encryption algorithm by using a shared secret key. Therefore, only parties that know the shared secret can decrypt it. In our scheme, only the access point stores that shared secret and can obtain the plain data. So, even though an adversary of **Type 1** eavesdrops on the transmission between sensor nodes and CH or the transmission between CH and access point, he cannot reveal the content of the messages. This way, the scheme provides end-to-end confidentiality. □

**Theorem** **2.** *Our scheme provides end-to-end integrity in the presence of an adversary of **Type 2***.

**Proof** **of** **Theorem** **2.** The scheme introduces a randomized watermarking technique that checks and verifies the integrity of all packets sent through the network. Any malicious act done to the packet will result in the rejection of that packet. In the following, we state how the scheme secures the network from an attack of the adversary of **Type 2**. □

(a) **Injection Attack:** an adversary needs to generate a valid watermark to embed it into the injected data. This requires him to know the shared secret cluster key to generate it. Even if the shared secret is somehow disclosed, he will then need to know the random places to embed it. This requires knowledge of the secret PRNG parameters. This way, all injected false data are rejected either at the CH or the access point.
(b) **Replay Attack:** an adversary resends old messages that he captures from the past. This will result in message drops, as all packets contain a timestamp to ensure that the packet is fresh.
(c) **Modification Attack:** an attacker can modify the data sent and the packet will still look valid. That is due to the homomorphic encryption function’s properties. For example, a message m can be altered to m+x, where *x* is the modified value. The recipient can decrypt the message and obtain the modified value. In our scheme, the watermark is computed from the encrypted message and embedded inside the payload. So, any alteration done to the encrypted data will result in a failure of verification of the watermark.

**Theorem** **3.** *Our scheme is robust against an internal adversary that can compromise a node even if he can control a node or CH from the network*.

**Proof**  **of** **Theorem** **3.** In the proposed scheme, each cluster has its own security parameter. Therefore, even when an adversary compromises a sensor node or a CH, he will be able to manipulate and control one cluster alone. The access point verifies all clusters’ data individually, and accepts the valid ones and rejects the others. □

**Theorem** **4.** *Our scheme provides en-route filtering for data injected into the network*.

**Proof** **of** **Theorem** **4.** The scheme verifies every packet from sensor nodes at CH. Therefore, any injected data at any level will be filtered at the next highest level. The end-to-end integrity that is provided by other schemes is not sufficient and can waste energy when forwarding false packets to the access point without verifying its integrity. □

## 6. Simulation and Results

In this section, we evaluate the performance of the RWFS scheme against the work presented by Cui et al. in [22]. We compare their performance regarding filtering capacity, average energy consumption, network lifetime, network overhead, and delay.

The simulations of both schemes were done using MATLAB. A heterogeneous WSN containing of n=100 nodes is considered in this simulation. The value of m = 0.1, which means that 10% of the total number of nodes are advanced nodes containing a times more energy than the normal nodes. The nodes were randomly deployed in a 100 m × 100 m topological area with one access point located at the center of the area (50 m,50 m). To unify the simulation of the two schemes, we used the same clustering algorithm (E-DEEC) [37]. Table 2 shows all of the parameters that were used in this simulation.

### 6.1. Filtering Efficiency

**Figure 6:**
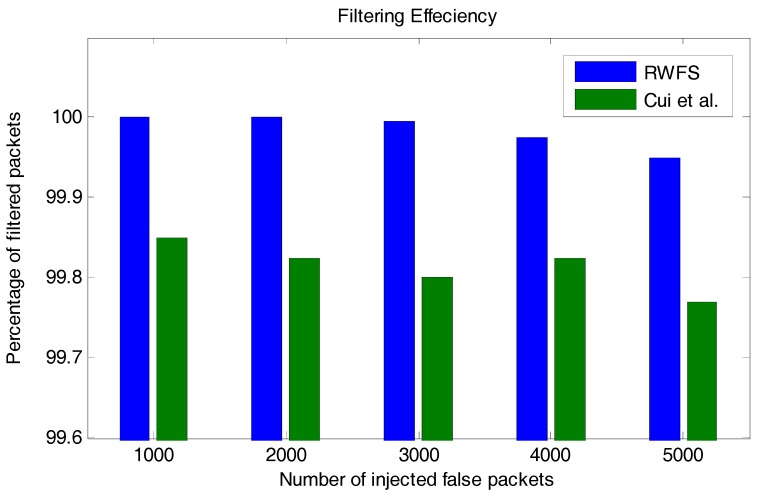
The filtering efficiency analysis between RWFS (the proposed scheme) and the Cui et al. scheme. The bar graphs show the percentage of filtered malicious packets for a different scenario where a different number of false packets was injected.

Filtering efficiency evaluates the ratio of the number of detected malicious packets to the total number of malicious packets. In Figure 6, we have compared the filtering efficiency between the new proposed scheme (RWFS) and the Cui et al. scheme in [22].

We have assumed that the attacker knows the MAC method that was used to generate the watermark in RWFS and the MAC method that was used to generate the MAC in the Cui et al. scheme. Therefore, the attacker can only randomize values that are secret parameters of each scheme.

For this factor, we have injected the network with different numbers of malicious packets and wanted to know how many packets were detected and rejected by the scheme. From the results in Figure 6, we can see that our scheme is better, with an accuracy of 99.95% rejection in the case of injecting 5000 malicious packets against 99.77% for the Cui et al. scheme. This percentage of filtering efficiency proves that the scheme can perform well against any number of injected packets.

### 6.2. Network Overhead

To achieve a fair comparison between our scheme and the Cui et al. scheme [22], we have unified the sizes of common parameters. We have assumed that packets sent during the “Setup and Key Management” phase in RWFS and the ones sent during the “Setup” phase in the Cui et al. scheme are both of equal sizes (4000 bits). In addition, both sensor nodes’ identities and timestamps were set to 8 and 64 bits, respectively.

In RWFS, a packet contains a cipher-text $c_{i}$ of size 16 bits, an embedded watermark of size 12 bits, the node’s identifier, and a timestamp, which makes a total of 100 bits of payload per packet. In the Cui et al. scheme, the packet contains a cipher-text $c_{i}$ of size 16 bits, a 48-bit homomorphic MAC, a MAC of size 160 bits, a node’s identifier, and a timestamp. This makes the size of a packet to be 304 bits.

**Figure 7:**
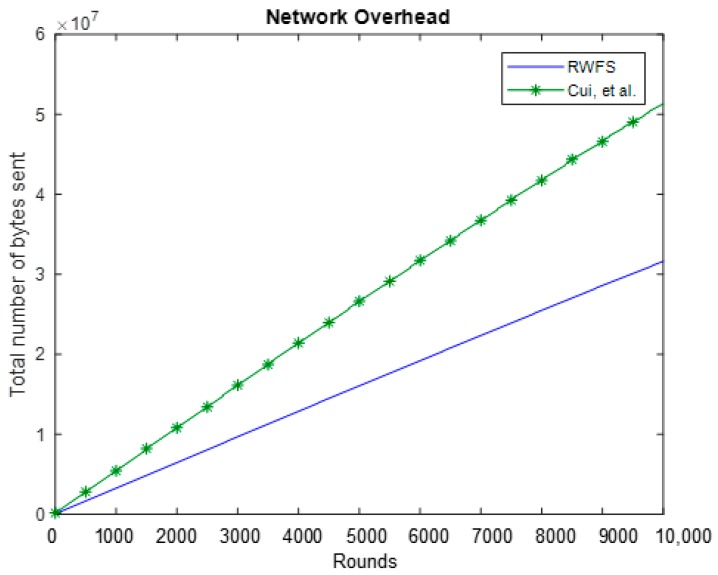
The network overhead analysis between RWFS (the proposed scheme) and the Cui et al. scheme. The two graphs show the total number of packets sent during 10,000 rounds.

The increase of the packet size is clearly reflected in the overall network overhead. From the comparison results in Figure 7, we can find that our scheme provides a great reduction in network overhead compared to the results of Cui et al.’s work for a duration of 10,000 rounds. The Cui et al. scheme transmits a total of 60% more bytes than our scheme. The reduction in transmitted packets can be directly linked to a longer network lifetime.

### 6.3. Average Energy Consumption

**Figure 8:**
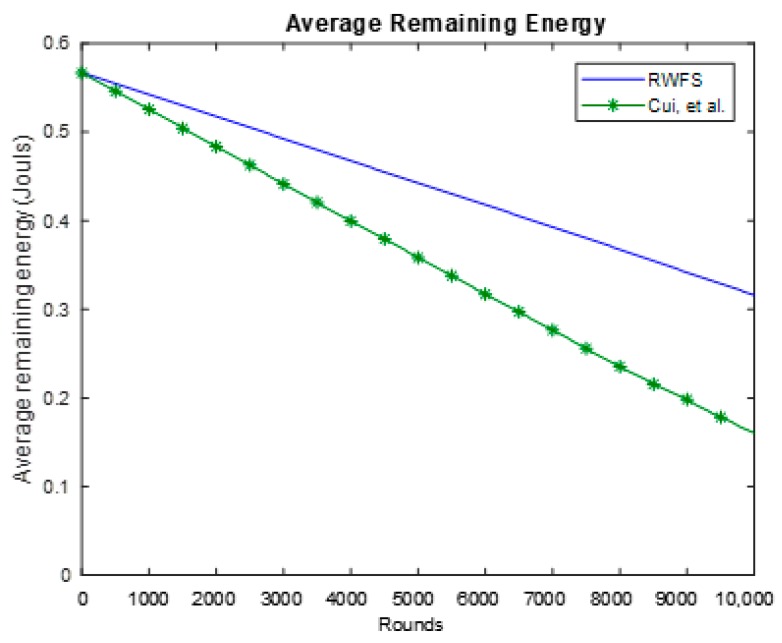
The average remaining energy analysis between RWFS (the proposed scheme) and the Cui et al. scheme. The average energy of all nodes in the network is calculated at each round for a duration of 10,000 rounds.

Energy consumption is one of the main factors that matter when developing a new protocol or scheme for WSNs. To evaluate this factor, we have computed the average remaining energy of the whole network after each round. The results are shown in Figure 8.

From Figure 8, we can see that our scheme performs better in terms of saving the energy of the network. RWFS can save as up to 85% more energy than the Cui et al. scheme. This is a direct impact of reducing the packet size. The larger the packet, the more energy is consumed by a node to send and receive packets.

### 6.4. Network Lifetime

**Figure 9:**
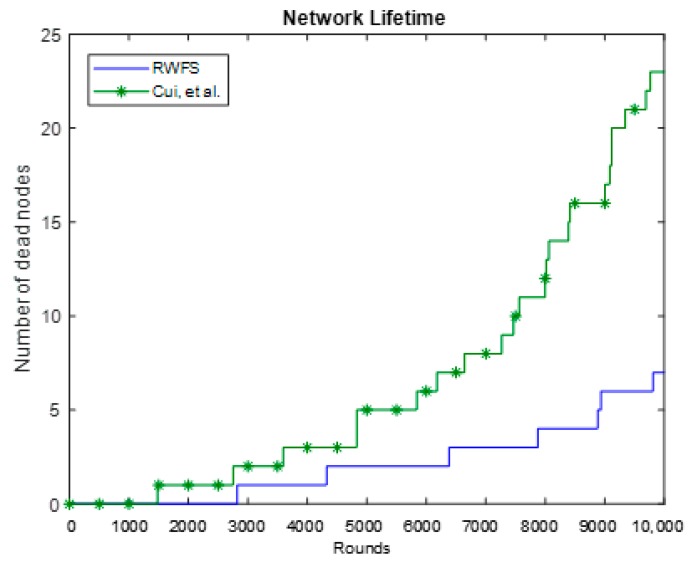
The network lifetime analysis between RWFS (the proposed scheme) and the Cui et al. scheme. The number of dead nodes is calculated at each round in a duration of 10,000 rounds.

Network lifetime can be defined as “*the time at which the first network node runs out of energy to send a packet*” [20]. Therefore, we have investigated the number of dead nodes after a number of rounds to get a clear indication of the network lifetime of both schemes. Figure 9 shows that, after 1500 rounds, a node was out of energy in the Cui et al. scheme, whereas RWFS reached 2800 rounds before the first node died. We can also see that, after 10,000 rounds, only seven nodes had died in our scheme, which is one-third of the number of dead nodes that were in the Cui et al. scheme.

This implies that our scheme is more energy-efficient than the one presented by Cui et al. This can be due to the fact that our scheme consumes less energy per round and uses a smaller packet.

### 6.5. Delay

We define delay here as the required time to complete a whole round. It comprises the time taken to sense and process data at the leaf nodes, verify and aggregate data at the cluster heads, and finally verify and decrypt data at the access point.

**Figure 10:**
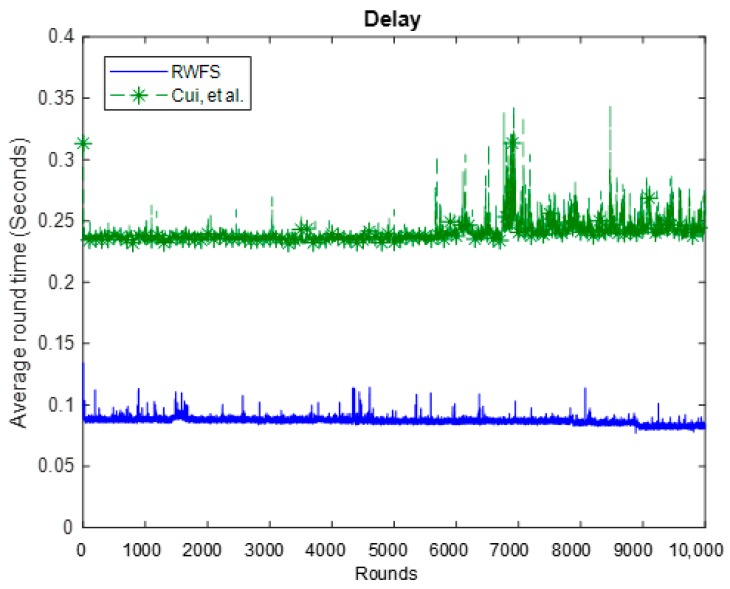
The delay analysis between RWFS (the proposed scheme) and the Cui et al. scheme. The average execution time for each round was measured for a total of 10,000 rounds.

From the results in Figure 10, we can see that the average delay of one round of RWFS is 0.086 s, which is 65% less than the delay of the Cui et al. scheme. This can be explained by the Cui et al. scheme having an additional homomorphic MAC (H-MAC) that is computed for each sensed data, along with a MAC. One calculation of the H-MAC alone was found to be 1.3 ms on average. This makes RWFS 1.3×n ms faster than the Cui et al. scheme.

## 7. Conclusions

IoT applications play a major role in building new solutions for various real-world problems. They connect physical sensors, field control centers, and cloud systems to enable reliable and smart applications. Recently, IoT applications have been experiencing tremendous industrial interest due to their advantages. However, physical attacks, such as false injection attacks, are considered to be one of the main threats for the security of the devices in the Internet of Things. False data injection attacks are considered to be very crucial in sensitive networks where any small alteration in the data measurement could lead to a severe consequence. Therefore, it is crucial to employ a scheme that provides a filtering ability against false data injection attacks, taking into consideration all of the limitations of the sensors that comprise the networked system. Several attempts have been made to design such a scheme, but all of them face some challenges. The common limitation of the existing solutions is energy-related issues.

This paper presented a lightweight, randomized watermarking filtering scheme (RWFS) that provides data integrity, along with data confidentiality, in a wireless sensor network for IoT applications. The primary goal of this proposed scheme is to be an energy-efficient mechanism for securing such networks against data injection attacks. We employ a homomorphic encryption algorithm to protect end-to-end data confidentiality and a watermark to achieve en-route data filtering. The scheme uses a watermark that is generated and embedded into the original data sent by all source nodes in the network. The watermark is generated based on four features, including an encrypted payload, the secret shared key of the cluster, the sensor’s identifier, and the data capture time of the individual sensor node. This watermark is then embedded into random places within the packet. These random places are generated using a PRNG function. After this, the watermarks are verified by the respective cluster head to ensure the integrity of the packets. For a testing purpose, we have simulated our scheme in comparison with the Cui et al. scheme. Cui et al.’s work uses a homomorphic MAC function to provide for end-to-end integrity, which is computationally expensive and takes more time during generation. Our experimental results proved that RWFS achieves better computational efficiency and consumes less energy.

## Figures and Tables

**Table 1:** System notation.

Notation	Description
$CH_{j}$	The cluster head node of the *j*th cluster in the network
$S_{i}$	The sensor node *i*
n	Number of sensor nodes per cluster
N	Number of sensor nodes in the whole network
$CH_{j}ID$	Cluster identifier of cluster $CH_{j}$
$K_{cj}$	The cluster key of the *j*th cluster in the network
$K_{m}$	The master key of the network
$W_{i}$	Watermark of the sensor node $S_{i}$
$D_{i}$	Sensing data of the sensor node $S_{i}$
$E_{i}$	Encrypted sensed data of the sensor node $S_{i}$
$R_{i}$	The complete report of the sensor node $S_{i}$

**Table 2:** Simulation Parameters.

Parameter	Value
Topographical Area	100 m × 100 m
Access point location	(50, 50)
Number of nodes	100
Fraction of advanced nodes (*m*)	*m* = 0.1
Initial energy of normal node ($E_{0})$	$E_{0}$= 0.5 J
$E_{elec}$	50 nJ/bit
$E_{fs}$	10 pJ/bit/m^2^
$E_{mp}$	0.0013 pJ/bit/m^2^
$E_{DA}$	5 nJ/bit
Packet header size	150 bits
